# Supplementary material for: Uneven economic burden of non-communicable diseases among Indian households: A comparative analysis
Source: PLoS One. 2021 Dec 10;16(12):e0260628. doi: 10.1371/journal.pone.0260628 (PMC8664228; doi:10.1371/journal.pone.0260628)
Supplement: S1 Appendix — (DOCX) [file pone.0260628.s001.docx]

**Annexure 1: Out-of-pocket expenditure as a proportion of total medical expenditure, NSSO survey 2017-18**

| Medical expenditure | \| Public \|  \| \| --- \| --- \| \|  \|  \| | | Private | |
| --- | --- | --- | --- | --- | --- | --- | --- | --- |
|  | NCD | Non-NCD | NCD | Non-NCD |
| Doctor fee | 0.06 | 0.07 | 0.16 | 0.20 |
| medicine | 0.40 | 0.44 | 0.21 | 0.23 |
| Diagnostic test | 0.15 | 0.16 | 0.10 | 0.09 |
| Bed charge | 0.04 | 0.05 | 0.11 | 0.12 |
| Other med expenditure | 0.10 | 0.14 | 0.08 | 0.08 |

Source: Author’s estimation based on NSSO survey, 75^th^ round, 2017-18
