# Supplementary material for: Uneven economic burden of non-communicable diseases among Indian households: A comparative analysis
Source: PLoS One. 2021 Dec 10;16(12):e0260628. doi: 10.1371/journal.pone.0260628 (PMC8664228; doi:10.1371/journal.pone.0260628)
Supplement: Attachment — Submitted filename: reviwer comments.docx [file pone.0260628.s002.docx]

**Comments**

1. **Introduction:**

- There are some spelling mistakes.
- Some abbreviations are written without the whole word at first such as GDP in page 3
- On the other hand, some phrases are repeated and abbreviations should be used such as DALY use the abbreviation not the Disability Adjusted Life Years and United Nations Sustainable Development Goals in page 3.
- The estimated figure in page 3.. what figure?

2. **Study design:**

- An NCD should be a NCD.
- Use the abbreviation for non-communicable disease

3. **Results:**

- Table 1: I think a column for the total % should be added
- INR: I understood it is for Indian Rupee but the whole word was not written anywhere in the manuscript
- Table 3: I think you can test the significant difference between different variables and put the p value
- Comment for table 3 should be before the table.
- Table 4: how is the percentage calculated? It is not equal 100%
- Table 5: why there is mention for 10% significant? Mostly we use 1% or 5% only. 10% consider not-significant

4. **Discussion:**

- Why there is a table 6 in the discussion? It should be in the result section and it is not present in the manuscript at all.

5. **Conclusion:**

- Use abbreviations for non-communicable disease, out-of-pocket expenditure and catastrophic health expenditure

6. **Ethical consideration:**

- I don't understand why there is no ethical approval or statement?
